# Systematic review of the stage of innovation of biological mesh for complex or contaminated abdominal wall closure

**DOI:** 10.1002/bjs5.78

**Published:** 2018-06-14

**Authors:** S. K. Kamarajah, S. J. Chapman, J. Glasbey, D. Morton, N. Smart, T. Pinkney, A. Bhangu

**Affiliations:** ^1^ College of Medical and Dental Sciences University of Birmingham Birmingham UK; ^2^ Department of Colorectal Surgery, Queen Elizabeth Hospital University Hospitals Birmingham NHS Foundation Trust Birmingham UK; ^3^ Leeds Institute of Biomedical and Clinical Sciences University of Leeds Leeds UK; ^4^ Exeter Surgical Health Services Research Unit Royal Devon and Exeter Hospital Exeter UK

## Abstract

**Background:**

Achieving stable closure of complex or contaminated abdominal wall incisions remains challenging. This study aimed to characterize the stage of innovation for biological mesh devices used during complex abdominal wall reconstruction and to evaluate the quality of current evidence.

**Methods:**

A systematic review was performed of published and ongoing studies between January 2000 and September 2017. Eligible studies were those where a biological mesh was used to support fascial closure, either prophylactically after midline laparotomy, or for reinforcement after repair of incisional hernia with midline incision. The primary outcome measure was the IDEAL framework stage of innovation. The key secondary outcome measure was the GRADE criteria for study quality.

**Results:**

Thirty‐five studies including 2681 patients were included. Four studies considered mesh prophylaxis, 23 considered hernia repair, and eight reported on both. There was one published randomized trial (IDEAL stage 3), none of which was of high quality; the others were non‐randomized studies (IDEAL stage 2a). A detailed description of surgical technique was provided in most studies (27 of 35); however, no study reported outcomes according to the European Hernia Society consensus statement and only two described quality control of surgical technique during the study. From 21 ongoing randomized trials and observational studies, 11 considered repair of incisional hernia and 10 considered prophylaxis (seven in elective settings).

**Conclusion:**

The evidence base for biological mesh is limited, and better reporting and quality control of surgical techniques are needed. Although results of ongoing trials over the next decade will improve the evidence base, further study is required in the emergency and contaminated settings.

## Introduction

Incisional hernias carry a significant burden for both patients and the health service^1^, ^2^, ^3^, ^4^. They prevent return to normal activities and can be painful. Elective repair can be challenging, and emergency repair carries significant clinical risks. Incisional hernia is common, occurring in up to 50 per cent of patients after laparotomy^5^ ^6^, and with the growing number of emergency laparotomies performed in the UK, the number of affected patients is likely to increase^7^.

To limit the number of incisional hernias there has been a focus on the use of prophylactic mesh reinforcement. The cost of mesh is far less than that of major reoperations and emergency admissions^3^ ^8^, ^9^, ^10^. Although synthetic meshes are accepted in many cases, they are not used in complex and contaminated settings owing to the risk of infection (as high as 50–90 per cent), pain, fistulation and need for explantation^11^, ^12^, ^13^, ^14^. Biological mesh has evolved to fill this gap, with expected reduced rates of infection leading to safer prophylaxis. Current guidelines, including the Ventral Hernia Working Group expert consensus, and several systematic reviews recommend against the use of synthetic mesh when the risk of wound complications is high, such as in the presence of gross contamination; instead they advocate the use of a biological absorbable mesh^15^, ^16^, ^17^.

Biological mesh has entered widespread clinical practice, but the quality and scope of the evidence base for use in complex and contaminated abdominal wounds are unclear. This review aimed to determine the quality and stage of innovation of the evidence supporting biological mesh placement during abdominal wall reconstruction with primary fascial closure. The hypothesis was that the evidence base supporting biological mesh use is currently too limited to support routine clinical use outside clinical trials.

## Methods

### Search strategy

A systematic search of PubMed, EMBASE and the Cochrane Library between 1 January 2000 and 27 September 2017 was performed by two independent investigators. The http://clinicaltrials.gov database was also queried for ongoing studies. The search terms used were ‘laparotomy’, ‘mesh’, ‘biologic material’, ‘abdominal wall’, ‘hernia’, and ‘complications’, ‘contamination’, ‘infection’ or ‘surgical site infection’, individually or in combination. The ‘related articles’ function was used to broaden the search, and all citations were considered for relevance. A manual search of reference lists in recent reviews and eligible studies was also undertaken. This paper is reported according to the PRISMA guidelines^18^.

### Inclusion and exclusion criteria

Studies were included according to the following criteria: evaluation of the use of a xenograft biological mesh to support primary fascial closure of midline abdominal wounds or repair of incisional hernia with midline incision; study design was an RCT, prospective observational study, retrospective cohort study or case series; study included only patients aged 16 years or more.

The following exclusion criteria were employed: study design was a systematic review, meta‐analysis, letter, review, comment or conference abstract; fewer than five patients were included in the study; only synthetic mesh or composite meshes were evaluated; allograft or autograft meshes, including human‐derived acellular dermal matrix, were used (availability in Europe across the selected inclusion dates was low until recently, so reporting is likely to be incomplete); study reported bridging repairs (fascial closure not achieved), including studies where outcomes for fascial closure were not reported separately from bridging repair.

### Study outcome measures

The primary outcome measure was the stage of innovation, according to the IDEAL framework^19^. The level of evidence in the IDEAL staging system were 1 (case series with high risk of bias), 2a (cohort study), 2b (feasibility RCT), 3 (RCT) and 4 (high‐quality prospective registry with long‐term monitoring and low risk of bias). All assessments in the present study were carried out independently by two authors; disagreement was resolved by re‐examining the relevant article until consensus was achieved.

### Secondary outcome measures

The main secondary outcome measure was the quality of evidence assessed using the GRADE system^20^. In the GRADE approach, studies are categorized as of high (randomized trials or double‐upgraded observational studies), moderate (downgraded randomized trials or upgraded observational studies), low (double‐downgraded randomized trials or observational studies) and very low (triple‐downgraded randomized trials, downgraded observational studies or case series/case reports) quality. The other secondary outcome measures of interest were the numbers of studies reporting: outcomes according to the European Hernia Society consensus statement^21^, incidence of incisional hernia, surgical‐site infection (SSI) rate, and seroma.

### Data extraction

Data extracted included patient demographics, indications and type of biological mesh used. Studies were grouped into those examining prophylactic placement in primary closure of laparotomy only (prophylaxis), repair of incisional hernia only (reinforcement), or both (mixed). Descriptions of procedures performed were collected, including surgical technique, number of procedures previously performed by the surgeon, and monitoring of technique. Degree of contamination (clean‐contaminated, contaminated or dirty surgery) was defined according to the US Centers for Disease Control and Prevention (CDC) surgical wounds classification^22^, and the location of biological mesh placement was also evaluated, as either intraperitoneal (intraperitoneal, intraperitoneal onlay mesh, underlay, intra‐abdominal) or extraperitoneal (sublay, onlay, inlay, retromuscular, retrorectus, prefascial)^23^.

### Statistical analysis

Analysis was intended to be primarily descriptive in nature, with no need for modelling or multivariable analyses. Event rates are reported as percentages. Continuous variables were tested for normality.

## Results

**Figure 1:**
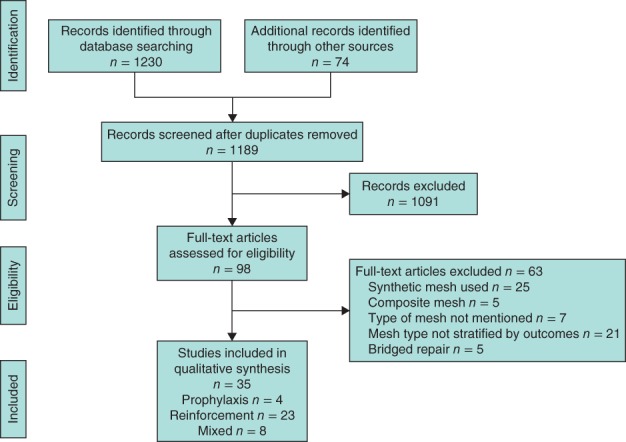
PRISMA diagram for the study

Of 1304 studies shortlisted, 35 full‐text articles^24^, ^25^, ^26^, ^27^, ^28^, ^29^, ^30^, ^31^, ^32^, ^33^, ^34^, ^35^, ^36^, ^37^, ^38^, ^39^, ^40^, ^41^, ^42^, ^43^, ^44^, ^45^, ^46^, ^47^, ^48^, ^49^, ^50^, ^51^, ^52^, ^53^, ^54^, ^55^, ^56^, ^57^, ^58^ met the inclusion criteria (*Fig*. 1). Of these, four examined biological mesh for prophylaxis, 23 reported on reinforcement after incisional hernia repair, and eight reported both prophylaxis and incisional hernia repair. Studies of biological mesh for prophylaxis included a total of 85 patients with a median follow‐up of 12 (i.q.r. 2–31) months; those used for reinforcement included 1744 patients with a median follow‐up of 16 (12–24) months, and those for mixed indications included 852 patients with a median follow‐up of 24 (17–48) months.

### Mesh characteristics

*Tables* 1 and 2 summarize characteristics of the included studies. Strattice™ (KCI Medical, Dublin, Ireland) (2 studies), Surgisis^®^ (Cook Biotech, West Lafayette, Indiana, USA) (1 study) and bovine pericardium (1) were used for prophylaxis in abdominal wound reconstruction. For reinforcement, Permacol™ (Tissue Science Laboratories, Andover, Massachusetts, USA) (9 studies) was the most commonly used mesh, followed by Strattice™ (4) and Surgisis^®^ (3); a further seven studies each used different meshes. In papers reporting mixed indications, Permacol™ (5 studies) was the most commonly reported, followed by XenMatrix™ (Brennen Medical, St Paul, Minnesota, USA; Davol, Warwick, Rhode Island, USA) (1), Strattice™ (1) and SurgiMend™ (TEI Biosciences, Boston, Massachusetts, USA) (1).

**Table 1:** Patient characteristics (arranged alphabetically by timing of surgery)

Reference	No. of patients	Median age (years)	Mean BMI (kg/m^2^)	Timing of surgery*	Indication for surgery
Bali *et al*.^26^	40	75	25	Elective	AAA repair
Bhangu *et al*.^25^	7	9	n.a.	Elective	Stoma closure
Boules *et al*.^49^	45	57	33	Elective	Incisional hernia repair
Boutros *et al*.^27^	8	60	n.a.	Elective	AWR after HIPEC
Chamieh *et al*.^31^	58	n.a.	n.a.	Elective	Incisional hernia repair
Chavarriaga *et al*.^32^	18	49	n.a.	Elective	Incisional hernia repair
Cheng *et al*.^28^	270	60	32	Elective	Incisional hernia repair
Cox *et al*.^48^	6	49	25	Elective	Incisional hernia repair
Fayezizadeh *et al*.^33^	77	56	35	Elective	Incisional hernia repair
Garvey *et al*.^52^	191	58	31	Elective	AWR, incisional hernia repair
Giordano *et al*.^47^	109	64	30	Elective	Incisional hernia repair
Giordano *et al*.^53^	484	59	31	Elective	Data not available
Gnaneswaran *et al*.^50^	12	51	32	Elective	Incisional hernia repair
Hicks *et al*.^34^	60	59	36	Elective	Incisional hernia repair
Høyrup *et al*.^57^	10	66	n.a.	Elective	Incisional hernia repair, stoma closure, left hemicolectomy, anterior resection, bowel obstruction
Hsu *et al*.^35^	28	55	34	Elective	Incisional hernia repair
Itani *et al*.^36^	80	57	n.a.	Elective	Incisional hernia repair
Limpert *et al*.^37^	26	54	n.a.	Elective	Incisional hernia repair
Madani *et al*.^38^	46	58	28	Elective	Incisional hernia repair
Maggiori *et al*.^24^	30	61	26	Elective	Stoma closure
Majumder *et al*.^39^	126	59	37	Elective	Incisional hernia repair
Nockolds *et al*.^40^	23	57	n.a.	Elective	Incisional hernia repair
O'Halloran *et al*.^41^	85	56	33	Elective	Incisional hernia repair
Patel *et al*.^42^	41	42	20	Elective	Incisional hernia repair
Rosen *et al*.^43^	128	58	34	Elective	Incisional hernia repair
Sbitany *et al*.^44^	41	66	25	Elective	Incisional hernia repair
Shah *et al*.^45^	58	57	34	Elective	Incisional hernia repair
Shaikh *et al*.^56^	20	51	n.a.	Elective	Incisional hernia repair, re‐exploration laparotomy, multiple stab wounds, desmoid tumour resection
Ueno *et al*.^29^	20	60	n.a.	Elective	Incisional hernia repair
Warwick *et al*.^30^	57	64	30	Elective	Incisional hernia repair
Zerbib *et al*.^46^	14	60	35	Elective	Incisional hernia repair
Abdelfatah *et al*.^58^	65	55	35	Mixed	Incisional hernia repair, intestinal obstruction (bowel strangulation and resection), resection of large section abdominal wall, infected alloplastic mesh
Byrnes *et al*.^51^	57	49	32	Mixed	Incisional hernia repair, trauma laparotomy
Parker *et al*.^54^	9	58	n.a.	Mixed	Incisional hernia repair, AWR for abdominal wall tumour
Pomahac and Aflaki^55^	16	59	28	Mixed	Incisional hernia repair, intra‐abdominal emergencies, extensive bowel resection, abdominal compartment syndrome secondary to necrotizing fasciitis

* Mixed indicates both elective and emergency surgery.

AAA, abdominal aortic aneurysm; AWR, abdominal wall reconstruction; HIPEC, hyperthermic intraperitoneal chemotherapy.

**Table 2:** Summary of surgery and mesh characteristics (arranged chronologically by indication)

Reference	Year	Country	Indication	Type of mesh	Median follow‐up (months)
Boutros *et al*.^27^	2010	USA	Prophylaxis	Surgisis®	6
Bhangu *et al*.^25^	2014	UK	Prophylaxis	Strattice™	1
Bali *et al*.^26^	2015	Greece	Prophylaxis	Bovine pericardium	36
Maggiori *et al*.^24^	2015	France	Prophylaxis	Strattice™	17
Ueno *et al*.^29^	2004	USA	Reinforcement	Surgisis®	16
Limpert *et al*.^37^	2009	USA	Reinforcement	Bovine pericardium	22
Hsu *et al*.^35^	2009	USA	Reinforcement	Permacol™	16
Chavarriaga *et al*.^32^	2010	USA	Reinforcement	Permacol™	7
Cox *et al*.^48^	2010	USA	Reinforcement	Surgisis®	10
Shah *et al*.^45^	2011	USA	Reinforcement	XenMatrix™	12
Patel *et al*.^42^	2012	USA	Reinforcement	Strattice™	16
Itani *et al*.^36^	2012	USA	Reinforcement	Strattice™	24
Rosen *et al*.^43^	2013	USA	Reinforcement	Strattice™	22
Nockolds *et al*.^40^	2014	UK	Reinforcement	Permacol™	17
Cheng *et al*.^28^	2014	USA	Reinforcement	Permacol™/Strattice™	25
O'Halloran *et al*.^41^	2014	USA	Reinforcement	Unknown	14
Zerbib *et al*.^46^	2015	France	Reinforcement	Permacol™	13
Giordano *et al*.^47^	2015	UK	Reinforcement	Permacol™	24
Sbitany *et al*.^44^	2015	USA	Reinforcement	Strattice™	5
Gnaneswaran *et al*.^50^	2016	Australia	Reinforcement	BioDesign®	14
Fayezizadeh *et al*.^33^	2016	USA	Reinforcement	Permacol™	28
Majumder *et al*.^39^	2016	USA	Reinforcement	Permacol™	22
Hicks *et al*.^34^	2016	USA	Reinforcement	SurgiMend™	12
Warwick *et al*.^30^	2017	UK	Reinforcement	Permacol™	18
Madani *et al*.^38^	2017	Canada	Reinforcement	Surgisis®	47
Chamieh *et al*.^31^	2017	USA	Reinforcement	Mixed	11
Boules *et al*.^49^	2018	USA	Reinforcement	Permacol™	72
Parker *et al*.^54^	2006	USA	Mixed	Permacol™	18
Shaikh *et al*.^56^	2007	Ireland	Mixed	Permacol™	18
Pomahac and Aflaki^55^	2010	USA	Mixed	Permacol™	17
Byrnes *et al*.^51^	2011	USA	Mixed	XenMatrix™	31
Høyrup *et al*.^57^	2012	Denmark	Mixed	Permacol™	8
Abdelfatah *et al*.^58^	2015	USA	Mixed	Permacol™	60
Garvey *et al*.^52^	2017	USA	Mixed	Strattice™	53
Giordano *et al*.^53^	2017	USA	Mixed	SurgiMend™	31

### IDEAL stage of innovation and GRADE quality of evidence

Distribution of IDEAL stage and GRADE quality of included studies are presented in *Tables* 3 and 4 respectively. Of the four prophylaxis studies, two^24^ ^25^ evaluated biological mesh at the time of stoma closure, one^26^ following midline laparotomy after abdominal aortic aneurysm (AAA) repair, and one^27^ after cytoreduction and hyperthermic intraperitoneal chemotherapy. All four studies included only elective patients and the degrees of contamination were clean‐contaminated (2) and contaminated (2). Strattice™ was used in two studies^24^ ^25^ with an intraperitoneal placement; the others used bovine pericardium in an extraperitoneal position (1)^26^ or Surgisis^®^ in an intraperitoneal position (1)^27^. One study^24^ was IDEAL stage 2a (low quality) and the other^26^ was IDEAL stage 3 (moderate quality). Two studies^25^ ^27^ reported only outcomes of patients with biological mesh; both studies were IDEAL stage 2a (very low quality).

**Table 3:** Distribution of IDEAL stage of innovation, by indication

	IDEAL stage				
Indication	1 (case report)	2a (cohort study)	2b (feasibility RCT)	3 (RCT)	4 (registry)
Total (*n* = 35)	0	34	0	1	0
Prophylaxis (*n* = 4)	0	3	0	1	0
Reinforcement (*n* = 23)	0	23	0	0	0
Mixed (*n* = 8)	0	8	0	0	0

**Table 4:** Distribution of GRADE study quality, by indication

	GRADE quality			
Indication	High	Moderate	Low	Very low
Total (*n* = 35)	0	5	18	12
Prophylaxis (*n* = 4)	0	1	1	2
Reinforcement (*n* = 23)	0	2	14	7
Mixed (*n* = 8)	0	2	3	3

Of the 23 studies^28^, ^29^, ^30^, ^31^, ^32^, ^33^, ^34^, ^35^, ^36^, ^37^, ^38^, ^39^, ^40^, ^41^, ^42^, ^43^, ^44^, ^45^, ^46^, ^47^, ^48^, ^49^, ^50^ using biological mesh for reinforcement, all reported only elective patients undergoing repair of incisional hernia. The degree of contamination in all studies was clean‐contaminated. Mesh placement was reported as intraperitoneal in five studies^34^ ^35^, ^42^ ^44^, ^46^, extraperitoneal in seven^30^ ^32^, ^33^ ^37^, ^40^ ^48^, ^50^ and a combination in ten studies^29^ ^31^, ^36^ ^38^, ^39^ ^41^, ^43^ ^45^, ^47^ ^49^. One study^28^ did not report the location of mesh placement. Four^30^ ^31^, ^39^ ^41^ of the 23 studies compared biological *versus* synthetic mesh. All 23 studies were IDEAL stage 2a (cohort studies). Seven^29^ ^31^, ^38^ ^40^, ^48^, ^49^, ^50^ were of very low quality, 14^30^ ^32^, ^33^, ^34^, ^35^, ^36^, ^37^ ^39^, ^42^, ^43^, ^44^, ^45^, ^46^, ^47^ of low quality, and two^28^ ^41^ of moderate quality. None reported standardization of technique or location of biological mesh placement; the choice of mesh type was based on the preference of operating surgeon.

Of the eight studies evaluating biological mesh for mixed indications, four included patients undergoing elective surgery and the remaining four studies included both elective and emergency operations. The eight studies involved a mixture of procedures, with degree of contamination ranging from clean‐contaminated to dirty. Mesh placement was intraperitoneal in six studies^51^, ^52^, ^53^, ^54^, ^55^, ^56^, extraperitoneal in one study^57^, and a combination in one study^58^. All were IDEAL stage 2a (cohort studies). Evidence was of very low quality in three studies^54^ ^56^, ^57^, low quality in three^51^ ^52^, ^55^, and moderate quality in two^53^ ^58^. The evidence in one study^58^ of abdominal wall reconstruction with porcine acellular dermal matrix (Permacol™) was of moderate quality owing to reporting of long‐term outcomes of at least 5 years.

### Outcome reporting

None of the studies in this review reported outcomes according to the European Hernia Society consensus statement^21^, and none reported ‘free from hernia’ survival times. All four studies^24^, ^25^, ^26^, ^27^ in the prophylaxis group reported a definition for detection of incisional hernia, which included a combination of clinical examination and radiological assessment. In the reinforcement group, 13^29^ ^30^, ^32^ ^33^, ^35^ ^36^, ^38^ ^39^, ^41^, ^42^, ^43^, ^44^ ^47^ of the 23 studies gave a definition for recurrence of hernia (6 clinical, 7 radiological, none patient‐reported). SSI rates were reported in one^25^ of the four studies in the prophylaxis group, and in 21 of the 23 studies in the reinforcement group. The incidence of seroma was reported in three prophylaxis and 19 reinforcement studies.

### Reporting of surgical technique

Of the 35 studies, 27 provided details of surgical procedures: all four studies in the prophylaxis group, 16 in the reinforcement group, and seven in the mixed group (*Table* 5). Only one paper^46^ reported the minimum number of procedures performed by the operating surgeons as a requirement.

**Table 5:** Reporting of surgical technique

	All indications*	Prophylaxis only	Reinforcement only
No. of studies	35	4	23
Total no. of patients	2681	85	1744
Mesh type			
BioDesign^®^	1	0	1
Bovine pericardium	2	1	1
Mixed	1	0	1
Permacol™	12	0	9
Permacol™/Strattice™	1	0	1
Strattice™	7	2	4
SurgiMend™	2	0	1
Surgisis^®^	4	1	3
XenMatrix™	3	0	2
n.r.	1	0	1
Location of mesh placement			
Intraperitoneal (intraperitoneal underlay)	14	3	5
Extraperitoneal (sublay, onlay, inlay)	9	1	7
Mixed (intraperitoneal and extraperitoneal)	11	0	10
n.r.	1	0	1
Description of procedure			
Detailed surgical technique provided	27	4	16
Surgeon's no. of previous procedures provided	1	0	1
Monitoring of technique	2	0	2

* Includes prophylaxis only, reinforcement only, and mixed. n.r., Not reported.

### Ongoing studies

Twenty‐one ongoing studies were identified from http://clinicaltrials.gov, of which ten were for prophylaxis and 11 for reinforcement. In the prophylaxis group, all were RCTs; four had completed data collection, five were still recruiting, and one had terminated early. Patient groups being studied included emergency midline laparotomy (1 study), elective patients for AAA repair (1), midline laparotomy (1), contaminated abdominal wall defect (1, terminated), abdominoperineal resection (1) and stoma closure (5). Of these ten, the majority studied Strattice™ (4), followed by Permacol™ (1) and Surgisis^®^ (1). The type of biological mesh was not mentioned in the remaining four studies. In the 11 ongoing trials of reinforcement, nine were RCTs and two were cohort studies. Two studies (1 cohort study of Permacol™ and 1 RCT of XenMatrix™) were in follow‐up phase; the remainder were still recruiting patients.

## Discussion

This review identified that the evidence base for biological mesh in complex and contaminated settings is still evolving, and highlighted areas for improvement. At present, the quality of the evidence base is generally low, with a few exceptions. The majority of studies included in this review were IDEAL stage 1 or 2 (case series or cohort studies) with a low or very low GRADE quality of evidence, indicating that biological meshes remain in the early stages of evaluation and adoption. This is compounded by a wide variation in mesh types and mesh placement, with little control for surgical technique, making synthesis of evidence ineffective.

There are two key recommendations from the present study. First, the evidence base needs to be improved by testing the efficacy of biological mesh in randomized trials. This should include standardization of techniques and reporting, and inclusion of more emergency cases to establish the limits of indication. Second, future studies should allow consistent reporting of mesh type and exact placement to enable high‐quality recommendations to help standardize practice. Until such data are available, use in selected higher‐risk patients (such as prophylaxis during abdominal wall closure in contaminated cases at high risk of incisional hernia) should be supported by data capture within controlled trials or registries. Routine clinical use in low‐risk patients is not yet justified.

Surgeons and patients will benefit from knowing about mesh performance based on the specific type of mesh, the position it is placed in, and the expected long‐term outcome. The present study identified variation in outcome reporting for recurrence rates, SSI and seroma. This variation precludes reliable assessment of outcomes and formation of recommendations. Recently, Blencowe and colleagues^59^ proposed a standard approach for the description, standardization and monitoring of the intervention to enable reliable assessment of outcome from this type of study and, importantly, reproducibility of an intervention by surgeons in their clinical practice. In this review, only one study^46^ had monitoring of technique by a senior surgeon to allow consistency of mesh placement.

It is plausible that different biological meshes may have varying failure rates, degrees of immunogenicity, biocompatibility and risk profiles^60^. In a rat study^61^ of 85 laparoscopic ventral hernia repairs, Strattice™ and Parietex™ (Covidien Surgical, Dublin, Ireland) were seen to grow a new mesothelial layer on their visceral side, whereas microscopic degradation and new collagen formation were seen in the Surgisis^®^ group. In a mouse model of 135 mice with peritonitis, XCM BIOLOGIC^®^ (LifeCell, KCI, Branchburg, New Jersey, USA) and Permacol™ showed better incorporation than Strattice™, whereas Strattice™ had fewer strong adhesions^62^. More accurate information from human studies may allow improved selection of mesh for patients in future clinical practice.

The direct advantages of biological mesh remain unproven in widespread practice. First, the long‐term durability of biological grafts used for complex abdominal wall reconstruction has been disappointing^36^ ^43^. Rosen and co‐workers^43^ reported the overall hernia recurrence rate as 31 per cent over a mean follow‐up of 21·7 (range 1–74) months, and estimated the 3‐year recurrence‐free survival rate to be 51 per cent. Second, implementation and use of biological mesh in clinical practice depend on the cost, as biological meshes can be up to ten times more expensive than synthetic ones^17^ ^63^. Totten *et al*.^64^ demonstrated that use of biological mesh for hernia repair can cost $21 000 (€17 100; exchange rate 20 April 2018) in comparison with synthetic mesh, which costs $7100 (€5780) for minimal improvement in surgical outcomes such as SSI.

With high costs of abdominal wall reconstruction using biological meshes and limited long‐term data, there has been emerging interest in the use of long‐term absorbable synthetic materials. These biosynthetic meshes are a clinical alternative to biological meshes and are significantly cheaper. A prospective longitudinal study by Rosen and colleagues^65^, evaluating the use of GORE^®^ BIO‐A^®^ (W. L. Gore, Newark, Delaware, USA) biosynthetic mesh in CDC class II–IV wounds, demonstrated an SSI rate of 18 per cent and a hernia recurrence rate of 17 per cent at 24 months. In contrast, the RICH trial^36^, which evaluated CDC II–IV wounds with biological mesh, had an SSI rate of 66 per cent and recurrence rate of 28 per cent at 24 months. Although this evidence with biosynthetic meshes is promising, any superiority over biological mesh in clean, clean‐contaminated, contaminated or infected wounds remains to be tested in RCTs.

Several ongoing cohort studies and RCTs will improve the evidence base, although they predominantly involve elective patients. Only three studies include both elective and emergency patients for prophylaxis. Future studies in high‐risk patients (such as those undergoing emergency surgery, with active sepsis or high BMI) will establish new indications for biological mesh, with potentially greater benefit in these patients. Preventing the need for reoperation in high‐risk groups is likely to provide even greater cost savings to health services.

There are weaknesses to this study. Assessment of quality using the GRADE tool is subjective, although this was overcome by discussion between the two authors involved in assessing grade of evidence, and resolving disagreement by re‐examining the relevant article until consensus had been achieved. Nevertheless, this scoring system is used widely for assessing strength of evidence in the literature^20^. Biosynthetic resorbable meshes and patients undergoing bridged repairs were not included in the study, as they represent a clinically separate group and are likely to have a different stage of innovation due to timing of introduction.

The evidence base for biological mesh in this clinical context is limited and evolving. Better reporting and quality control of surgical techniques is needed and, although new trial results over the next decade will improve the evidence base, more trials in emergency and contaminated settings are required.

## Disclosure

The authors declare no conflict of interest.
